# Evidence-based de-implementation for contradicted, unproven, and aspiring healthcare practices

**DOI:** 10.1186/1748-5908-9-1

**Published:** 2014-01-08

**Authors:** Vinay Prasad, John PA Ioannidis

**Affiliations:** 1 National Cancer Institute, Bethesda, MD (VP) and Stanford Prevention Research Center, Departments of Medicine and Health Research and Policy, Stanford University School of Medicine, and Meta-Research Innovation Center at Stanford (METRICS), Medical School Office Building, Room X306, Stanford, CA 94305, USA

**Keywords:** Evidence-based medicine, Reversals, Divestment, De-implementation, Contradiction, Bias

## Abstract

Abandoning ineffective medical practices and mitigating the risks of untested practices are important for improving patient health and containing healthcare costs. Historically, this process has relied on the evidence base, societal values, cultural tensions, and political sway, but not necessarily in that order. We propose a conceptual framework to guide and prioritize this process, shifting emphasis toward the principles of evidence-based medicine, acknowledging that evidence may still be misinterpreted or distorted by recalcitrant proponents of entrenched practices and other biases.

## Background

Divesting from ineffective and harmful medical practices has the potential to improve outcomes for patients, and mitigate the unsustainable rise in healthcare costs. Abandonment (de-implementation) of medical interventions may depend on multiple factors. Empirical evidence from well-designed studies should count, but other considerations such as inertia, financial and professional conflicts, cultural and societal values, knowledge brokering, and lobbying may also be very important eventually. The question is how we can position evidence so as to be more informative and influential in these complex processes. Here we provide a framework to guide the evidence-based de-implementation of interventions, acknowledging how on-the-ground realities can enter these considerations. Broadly, we will consider three categories of healthcare practices: those that are known not to work; those for which the evidence base is uncertain; and those that are in development and where strategic preemptive placement of evidence may help their eventual de-implementation, if needed. While the examples herein draw upon our experience appraising medical practices, the principles are broadly applicable to all healthcare fields.

### Contradicted established medical practices

The number of medical practices where the best evidence shows no efficacy or harms outweighing benefits is substantial. One search produced over 150 potentially ineffective or unsafe practices [1], and empirical reviews of high impact medical journals have generated over 140 reversed medical practices [2].

When large, well-done randomized trials have contradicted current medical practice, de-implementation makes sense, but it can meet with fierce tactical resistance. Proponents of contradicted medical practices can procure not only editorials, but also counter-evidence that cuts corners, *e.g*. focusing on lesser endpoints, highlighting subgroup analyses, or performing additional studies with straw man controls. Expert-based meta-analyses with tailored eligibility criteria and outcome selection to show some benefit [3], and conflicted expert guidelines can follow suit [4].

Take for example the 2007 COURAGE study, which found that among patients with coronary artery disease and stable angina, routine percutaneous coronary intervention (PCI) was no better than an initial strategy of optimal medical treatment (OMT). In the month following the study’s publication, PCI and stenting was down 13% nationally; however, by 2010, those numbers returned to prepublication levels [5]. Not only has COURAGE and corroborating meta-analyses [6,7] failed to stem the use of PCI, but it appears that they have also not improved adherence to OMT at time of PCI [8]. Proponents of stenting criticized COURAGE, citing selection bias, crossover, and poor study power [9]. Then, in 2012, FAME 2 showed that PCI guided by fractional flow reserve testing could decrease rates of revascularization compared to OMT, though there were no differences in cardiac death, myocardial infarction, or stroke [10]. Yet the results of FAME 2 and COURAGE are comparable. Regarding revascularization, 10 stents were placed to avert 1 future revascularization procedure in FAME 2, and 12 stents achieved that goal in COURAGE. Whether PCI was guided by angiography or fractional flow, the net result is similar.

Moreover, multiple meta-analyses of PCI co-authored by interventional cardiologists and/or sponsored by the industry claimed benefits in PCI by pooling together trials of stable angina and trials with residual ischemia, transferring the benefits of the latter population to the former. Practice guidelines then could follow the same path.

Another example is the routine use of gown and glove precautions among patients colonized with resistant pathogens, which is supported only by quasi-experimental, before-and-after, studies [11]. Yet, to date, two cluster-randomized trials have failed to support the benefits of this practice [12,13]. One study showed no reduction in the transmission of methicillin-resistant Staphylococcus aureus or vancomycin-resistant enterococcus [13], and the other showed no difference in rates of colonization or infection with these two pathogens [12]. These studies have failed to change this practice, however, and editorial ambivalence continues [14]. Some have even claimed that ‘the likelihood of harm (more than clear evidence of benefit) should drive the decision to implement’ [15], *i.e*. considering de-implementation only after contact precautions are proven to be harmful, not merely ineffective. Such resistance to adhere to the best available evidence inflates healthcare costs, and may distract from alternative strategies with promising early results of efficacy, such as universal decolonization protocols [16].

In brief, evidence wars can hinder de-implementation, and ‘practice resuscitation’ may be successful at reclaiming lost market shares. Of course, it is entirely possible that some subgroup truly benefits when a practice has ‘negative’ results globally, or that some lesser endpoints are meaningful to patients. Yet, more often such counter-evidence resurrection studies simply create excuses to not abandon the contradicted practice.

We propose a simple standard to curb such practice resuscitation: The evidence to revive a contradicted medical practice (whether in part or in whole) should involve endpoints and controls at least as rigorous as the contradictory study. Until such evidence is obtained, payers may offer disincentives by placing restrictions to reimbursement, and regulators may consider revoking or restricting prior approvals.

### Unproven medical practices

Clearly contradicted practices are less common than unproven ones. Among 1,344 articles assessing a medical practice, 363 (27%) tested standard of care, with 146 (11% of the total) contradicting it [2]. Many medical practices are largely untested or have insufficient evidence. An empirical evaluation of the Cochrane Database of Systematic Reviews found that the existing evidence base was unable to support or refute 49% of interventions [17], and 48% of American College of Cardiology recommendations were supported by expert opinion only [18].

A rational strategy to de-implement medical practices supported by little to no evidence is to subject them to testing in systematic fashion. Ideally, this assessment would be performed under the auspices of non-conflicted bodies, possibly within existing governmental structures, such as the Agency for Healthcare Research and Quality. Table 1 highlights potential considerations to prioritize untested medical practices. Likely major considerations include the extant evidence base of a practice — preference should be given to those based on the least evidence — and the cost and ubiquity of the practice — preference given to those practices placing the largest burden on the healthcare system. Additional considerations include the presence of alternative choices in a field — preference given toward reappraising fields with many alternatives of varying class, price, and evidence base; practices with clearly documented harms; practices where the cost to obtain the necessary evidence is contained; and practices where the results of trials with unfavorable results may realistically change minds and practice. Formal approaches, such as value of information calculations [19] may be applied, investigating the value of specific proposed randomized trials towards de-implementing established unproven practices.

**Table 1:** Potential considerations in prioritizing the testing of unproven medical practices

**Factor to consider**	**General principle**	**How to implement this factor**
**Prior evidence base**	Priority should be given to practices where the present evidence base is weakest.	For instance, a tiered system may be utilized: Level 1 (Weak) Randomized trials of interventions claiming subjective benefits, that are unblinded or fail to use proper controls. 2 (Weaker) Historically controlled studies of interventions that purport survival benefits, case series documenting improvements in subjective endpoints and quasi-experimental studies. 3 (Weakest) Practices based on pathophysiology and expert opinion alone. In many cases, professional conflicts may also prove problematic; thus, it may be reasonable to pursue this technique using content-specific experts in strictly an advisory capacity
**Cost/ubiquity**	Priority should be given to interventions with significant net financial burden on health payers.	For instance, orthopedic procedures for chronic back and joint pain, including knee and hip replacement surgeries are widely utilized in the United States, incur large financial burden on payers, but have little evidence of sustained long term benefits.
**Alternative options**	Priority should be given to practices for which there are several alternative options, particularly if alternatives are of completely different mechanisms (thus unlikely to also be overturned), or of low cost or bolstered by stronger evidence.	For instance, consider the market for anti-rheumatologic agents. Maintenance treatment of rheumatoid arthritis (RA) with disease modifying agents (DMARDS) has historically relied upon oral anti-immunologic agents such as methotrexate, azathiaprine, cyclosporin, and hydroxychloroquine. Recent years have witnessed a boom in novel drugs, typically expensive monoclonal antibodies against circulating cytokines or cell surface receptors. To date, this market has been limited by paucity of head to head trials, and, of trials that have been conducted, the majority are industry-sponsored studies. Collectively, there remains clinical uncertainty about how best to use these agents [20].
**Documented harms**	Priority should be given to test practices where the harms are well documented and confer substantial morbidity.	For instance, there is growing awareness of strut fracturization, embolism, and migration of IVC filters. At the same time, the IVC filter has never shown to improve any patient-centered outcome for any patient population in a prospective trial, and traces its approval through the FDAs 510 k mechanism [21].
**Testing the intervention makes financial sense**	Priority should be given to test practices where the cost to test is far less than ongoing expenditures of the practice.	In some respects, trialists should think like CEOs, weighing the costs of conducting a study, which may find a practice ineffective versus the ongoing expenditures for that practice. At times, such calculations may favor costly trials where the existing evidence base is weak, observational studies suggest inefficiencies, and the ongoing costs are large [22]. At other times, small trials that eliminate boutique practices may be employed [23]. Whose financial bottom line is being affected is important to consider. For that reason, nonconflicted bodies should make these determinations, utilizing investigators without financial conflicts of interest.
**Proponents are open-minded**	Priority should be given to test practices where negative results may truly gain traction.	Some specialties (primary care providers) may be more ready to abandon contradicted medical practices, and it is reasonable to test practices when there is genuine belief that contradiction can gain traction. Furthermore, some practices may be cumbersome (tight glycemic control in the ICU), time-consuming (routine gown and glove precautions) or unpleasant, and their contradiction may also be palatable. Finally, as payment structures shift from fee for service towards bundles [24], costly components may lose faithful disciples. Other fields, those with numerous and hyperbolic third party advocates, have been notoriously unwilling to trust results that undermine their worldview, no matter how robust the science.
**Value of information gained**	Priority should be based on the expected value of funding a specific study that may inform de-implementation, at the size and cost proposed.	Value of information (VOI) offers a decision-making framework that tries to capture several of the above issues, at least the ones that can be best quantified [19]. VOI can be used to prioritize and power clinical trials taking into account the costs of increasing study sample size, the potential number of persons affected by changes in that practice, the costs of the practice, including downstream costs, and the increased knowledge of marginal changes in health outcomes that may result from testing — converting all to the final common denominator of cost per favorable outcome gained.

### Novel medical practices

With multiple novel interventions (therapeutic, diagnostic, prognostic, healthcare system, and other) being introduced in medical care, a key consideration is to take preemptive steps that would allow efficient de-implementation if the intervention eventually proves inefficient and harmful. While there is increasing pressure to adopt novel interventions before substantial evidence has been obtained on them, one method to curb the spread of ineffective practices is to restrict their use prior to widespread dissemination, as demonstrated by the case of percutaneous transluminal angioplasty and stenting (PTAS) for intracranial stenosis.

In 2005, the Wingspan intracranial artery stent was granted humanitarian device exemption from the US Food and Drug Administration, based upon provisional data that it could improve intracranial artery lumen diameter in patients with stenosis refractory to medical therapy [25]. However, the single, uncontrolled study that led to approval was unable to inform any patient-centered endpoint. In 2006, Centers for Medicare & Medicaid Services (CMS) announced that they would pay for the procedure only within the confines of a randomized trial. They adhered to such a position, despite pressure from the manufacturer in 2008 [26].

In 2011, the only randomized study of the device, the SAMMPRIS trial, found that PTAS among patients with a recent transient ischemic attack (TIA) or stroke and documented stenosis of a major intracranial artery nearly tripled the 30-day risk of stroke or death compared to optimal medical management (14.7% versus 5.8%) [27].

During the years it was approved but trial data was lacking, CMS’s policy dramatically limited off-protocol use of the device and effectively protected the public. Altogether, only a few hundred patients received the device (200+ treated on protocol) in the US [26] — contrast this against the millions of patients who received PCI for stable angina. CMS’s wise 2006 decision likely averted a catastrophic outcome for thousands of patients who might otherwise have been treated with the device.

The lesson of PTAS is that higher upfront standards have potential to protect patients from ultimately flawed care. Unfortunately, regulatory agencies appear to move increasingly in the opposite direction, notably with the creation of the FDA’s ‘breakthrough’ designation [28], and emerging guidance to industry for expanded options of accelerated approval [29,30] —a regulatory mechanism where developers have historically shirked post-marketing commitments of conducting trials examining clinically meaningful endpoints [31].

### Empirical testing

The opinions that we express here may well be biased. We believe, however, that there is no reason that experimental studies cannot be leveraged to provide clarity for health policies with broad societal repercussions. To date, regulatory policies have been based on theory or scant retrospective observational studies, but at least some policies may be tested creatively with randomized controlled trials [14]. For instance, novel agents may be randomly assigned to accelerated or traditional approval. This might help inform whether provisional approval, wide dissemination, and subsequent confirmatory trials benefit or harm society more than restrictive approval strategies sating robust endpoints prior to dissemination.

## Conclusion

De-implementing practices reflects a recommitment to evidence-based healthcare. This is important for medications, devices, procedures, behavioral or psychological interventions, screening and diagnostic tests, and any other intervention undertaken by people in the health professions. Strategies to eliminate ineffective and harmful practices may help contain healthcare spending and optimize outcomes. Ideally, the majority of medical decisions should be supported by robust data, with ambiguous decisions made only within the confines of ongoing studies. However, as we stated, rational, quantitative evidence may not necessarily be the only or even main factor driving healthcare decisions. Research to understand better the other, cognitive or political factors that facilitate or hinder de-implementation is thus also warranted (see Box 1).

## Box 1: Note from the editors

The Editors-in-Chief *of Implementation Science* invited this editorial following a consultation with our editorial team and Editorial Board. They identified “de-implementation“ as an important theme, which deserves more attention than it currently receives. We regard de-implementation broadly as “stopping practices that are not evidence-based”. We encourage further papers on this theme and will include these in a special article series in the journal to enhance their visibility. All submissions will be reviewed and handled according to our normal procedures. In addition, we welcome and encourage comments in response to the accompanying editorial, using the comment feature of the journal’s platform. These comments, while moderated, are intended to stimulate discussion and debate within the implementation research community. In both papers and comments, we welcome a range of perspectives and rigorous studies on the theme of de-implementation, including (but not limited to) contributions that cover psychological, organizational or economic factors. We intend to promote other themes in the future.

## Competing interests

The authors declare that they have no competing interests.

## Authors’ contributions

VP and JPAI drafted the manuscript. VP and JPAI edited the manuscript for intellectual content. JPAI provided supervision. Both authors read and approved the final manuscript.
